# Comparing Human Metapneumovirus and Respiratory Syncytial Virus: Viral Co-Detections, Genotypes and Risk Factors for Severe Disease

**DOI:** 10.1371/journal.pone.0170200

**Published:** 2017-01-17

**Authors:** Nina Moe, Sidsel Krokstad, Inger Heimdal Stenseng, Andreas Christensen, Lars Høsøien Skanke, Kari Ravndal Risnes, Svein Arne Nordbø, Henrik Døllner

**Affiliations:** 1 Department of Laboratory Medicine, Children’s and Women’s Health, Faculty of Medicine, Norwegian University of Science and Technology, Trondheim, Norway; 2 Department of Pediatrics, St. Olavs Hospital, Trondheim University Hospital, Trondheim, Norway; 3 Department of Medical Microbiology, St. Olavs Hospital, Trondheim University Hospital, Trondheim, Norway; Kliniken der Stadt Köln gGmbH, GERMANY

## Abstract

**Background:**

It is unclarified as to whether viral co-detection and human metapneumovirus (HMPV) genotypes relate to clinical manifestations in children with HMPV and lower respiratory tract infection (LRTI), and if the clinical course and risk factors for severe LRTI differ between HMPV and respiratory syncytial virus (RSV).

**Methods:**

We prospectively enrolled hospitalized children aged <16 years with LRTI from 2006 to 2015. Children were clinically examined, and nasopharyngeal aspirates were analyzed using semi-quantitative, real-time polymerase chain reaction tests for HMPV, RSV and 17 other pathogens. HMPV-positive samples were genotyped.

**Results:**

A total of 171 children had HMPV infection. HMPV-infected children with single virus (n = 106) and co-detections (n = 65) had similar clinical manifestations. No clinical differences were found between HMPV genotypes A (n = 67) and B (n = 80). The HMPV-infected children were older (median 17.2 months) than RSV-infected children (median 7.3 months, n = 859). Among single virus-infected children, no differences in age-adjusted LRTI diagnoses were found between HMPV and RSV. Age was an important factor for disease severity among single virus-infected children, where children <6 months old with HMPV had a milder disease than those with RSV, while in children 12–23 months old, the pattern was the opposite. In multivariable logistic regression analysis for each virus type, age ≥12 months (HMPV), and age <6 months (RSV), prematurity, ≥1 chronic disease and high viral loads of RSV, but not high HMPV viral loads, were risk factors for severe disease.

**Conclusions:**

Among hospitalized children with LRTI, HMPV manifests independently of viral co-detections and HMPV genotypes. Disease severity in HMPV- and RSV-infected children varies in relation to age. A history of prematurity and chronic disease increases the risk of severe LRTI among HMPV- and RSV-infected children.

## Introduction

Since the discovery of human metapneumovirus (HMPV) in 2001 [1], studies from all parts of the world have shown that HMPV causes respiratory tract infection (RTI) in children [2–7]. HMPV is usually detected in airway secretions from children with RTI by use of polymerase chain reaction (PCR) tests, and the virus seldom appears in healthy children [8]. HMPV has been classified into the *Pneumoviridae* family. Two main genotypes (A and B) and at least four genetic subtypes (A1, A2, B1 and B2) exist [9–12]. Whether these genotypes cause similar or different infections is largely unclarified because some studies have shown quite similar manifestations [13–15], whereas others found that either genotypes A [16,17] or B [18] may cause more severe disease.

Using sensitive nucleic-acid based molecular tests to diagnose viral pathogens has revealed that many children with LRTI have more than one virus present in the respiratory tract [14,19,20]. Previous studies in HMPV-infected children found that such viral co-detections were associated with increased disease severity [21,22]. However, this was not confirmed in other studies [14,17,19,23].

HMPV is closely related to respiratory syncytial virus (RSV), by far the most important airway virus affecting children worldwide [6,14,20,24]. It has been reported that RSV and HMPV infections in children may be quite similar [23,25,26], but there is also some evidence that RSV causes more severe disease than HMPV [6,18]. Risk factors for severe RSV infection are young age, prematurity, chronic lung disease, chronic heart disease and severe neurological disabilities [18,24,27–30]. Although it has not been studied to the same extent, it seems that some of these factors may also increase the risk of severe HMPV infection [2,17,18,29,31–33]. With some exceptions [18,29], most studies have separately dealt with risk factors for severe HMPV and RSV infections.

In the present study, we prospectively enrolled a large cohort of children <16 years old, who were admitted to hospital with acute RTI during a nearly 9-year long period from 2006 to 2015, and diagnosed a broad panel of respiratory viruses. Our primary aim was to study clinical manifestations in children with HMPV lower respiratory tract infection (LRTI), taking viral co-detections and HMPV genotypes into account. Our secondary aim was to compare HMPV- and RSV-infected children with LRTI, with a special emphasis on clinical manifestations and risk factors for severe disease.

## Materials and Methods

### Study Design and Population

**Fig 1:**
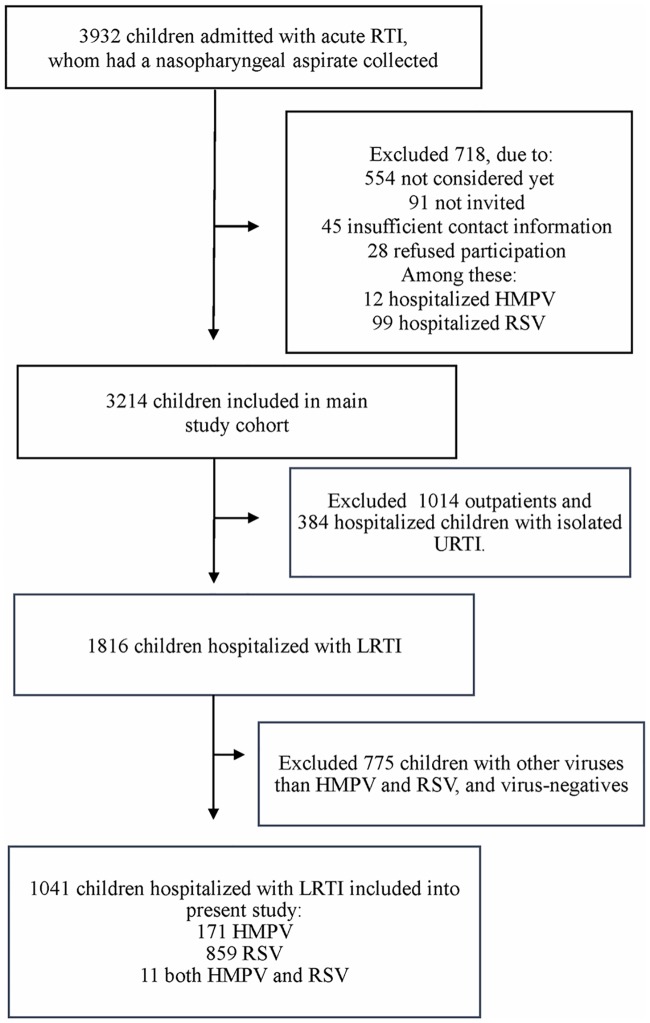
Study flow chart. Children were enrolled from the beginning of November 2006 to the end of July 2015. HMPV, human metapneumovirus; LRTI, lower respiratory tract infection; RSV, respiratory syncytial virus; RTI, respiratory tract infection; URTI, upper respiratory tract infection.

During the period from November 2006 to August 2015, we prospectively enrolled children aged <16 years who were admitted with acute RTI at the Pediatric Emergency Department, at the Department of Pediatrics, St. Olav’s Hospital, University Hospital of Trondheim, Norway, and who were sampled on clinical indications with a nasopharyngeal aspirate (Fig 1). The hospital provides care for 58,000 children younger than 16 years and 18,000 children younger than five years of age from Sør-Trøndelag County in Mid-Norway (Statistics Norway). Written informed consent to participate was collected from children ≥12 years and caregivers to most of the children during the hospital stay. However, due to practical challenges, including dealing with children 24 hours a day, we also enrolled some children after hospital discharge. Their caregivers received written information per regular mail, and the child was included if the caregivers did not resist enrollment by contacting the hospital within two weeks. The latter inclusion method was also approved by the Regional Committee for Medical and Health Research Ethics. Some children were included more than one time if they were hospitalized with distinct RTI episodes, whereas recurrent hospitalizations due to the same RTI were only registered once. Exclusion criteria were children evaluated for RTI who were hospitalized mainly due to other diseases, such as newborns not dismissed from the hospital, children with cytostatic or immune-suppressive treatment, and children with other infections such as gastroenteritis and urinary tract infection. We systematically collected baseline characteristics and past and current medical history from caregivers, who filled out a questionnaire, while clinical information was abstracted from medical records. Characteristics included gestational age at birth, age, gender, siblings, day care attendance and chronic diseases. Preterm birth was defined as a gestational age <36 weeks of gestation, and age was categorized according to clinically relevant groups. Chronic airway diseases, other than asthma, included bronchopulmonary dysplasia, congenital airway malformations, cystic fibrosis and neuromuscular disorders with hypoventilation in need of respiratory support and daily inhalations for mucus clearance. Tachypnea was defined as a ≥10 increase in normal respiratory rate according to the child’s age [34]. Several children had more than one chronic disease, i.e. both asthma and other chronic airway disease, or both cerebral palsy and epilepsy. Hence, for the logistic regression analyses, we used a combined binary variable: “≥1 chronic disease” (coded: no chronic disease or ≥1 chronic disease). Nasopharyngeal aspirates were collected within 24 hours of presentation in the vast majority of the children. Children with a stay <24 hours were categorized as outpatients, while children who stayed ≥24 hours and were admitted to the wards of the Pediatric Department, were categorized as inpatients. From the main cohort of children with acute RTI, we selectively included HMPV- and RSV-infected children hospitalized with LRTI into the present study (Fig 1). Consequently, outpatients, children admitted with isolated upper RTI (URTI) and children admitted with LRTI and viruses other than HMPV or RSV, and virus negatives, were excluded (Fig 1). The Regional Committee for Medical and Health Research Ethics approved the study.

### Clinical Investigations and Diagnostic Criteria

All children were routinely treated at the discretion of the medical doctors at the Department of Pediatrics. Children with one or more characteristic manifestations of rhino-pharyngitis, tonsillitis, conjunctivitis, otitis media or acute laryngitis were diagnosed with URTI. LRTI was categorized into five categories based on clinical manifestations and radiological findings: 1) Bronchiolitis: age <24.0 months old, tachypnea or signs of lower airways obstruction and a normal radiogram or a radiogram with air trapping/hyperinflation, perihilar infiltrates and/or atelectasis, 2) Obstructive bronchitis: age ≥24.0 months old, signs of lower airway obstruction and a normal radiogram or a radiogram with air trapping/hyperinflation, perihilar infiltrates and/or atelectasis, 3) Pneumonia: presence of dyspnea with or without auscultatory findings such as muffled respiratory sounds and a radiogram with localized or lobular infiltrates, or pleural effusion, 4) Asthma exacerbation: signs of lower airway obstruction and either a current asthma diagnosis or two or more previous episodes with lower airway obstruction during the previous year, or one or more episodes of lower airway obstruction and atopic status (eczema, allergy), and 5) Unspecified LRTI: cough or other signs of LRTI without signs of lower airway obstruction, with or without auscultatory findings and a normal radiogram or radiogram with perihilar infiltrates and/or atelectasis. Some asthmatic children who developed symptoms and signs of pneumonia were categorized with pneumonia.

### Respiratory Support and Disease Severity Measures

Respiratory support (RS) with a non-invasive ventilator (NIV) included positive airway pressure via mask in continuous or bi-level modes, or via a high-flow nasal cannula. Prior to 2011, all children in need of NIV were admitted to the Pediatric Intensive Care Unit (PICU). During 2011, respiratory support with high-flow nasal cannula was introduced at the wards, which reduced the need for PICU admissions. Invasive ventilator (IV) support was defined as RS applied by endotracheal tube or tracheostomy, and children with acute respiratory failure in need of IV were admitted to the PICU. Initiation of any RS, or an increase in baseline RS for those with chronic RS, were defined as RS related to the acute RTI. To adjust for our new practice performing NIV treatment at the wards, we defined a disease severity score reflecting disease severity independently of treatment location. This score, ranging from 0 to 4 points, was defined as the sum of: 1) a need for oxygen to maintain oxygen saturation ≥93% (1 point); 2) length of stay ≥6 days, corresponding to or above the 75 percentile limit for all hospitalized children with LRTI (1 point); 3) a need for respiratory support with NIV (1 point); 4) a need for respiratory support with both NIV and IV (2 points); and 5) need of IV support (2 points). In addition, admission to the PICU, was reported as a disease severity measure. Severe disease was defined as a disease severity score ≥2 points, corresponding to- or above the 75% percentile limit among all hospitalized children with LRTI.

### Laboratory Investigations

Nasopharyngeal aspirates (NPA) were collected and placed into a standard virus transport medium without antibiotics, and analyzed at the Department of Medical Microbiology, St. Olavs Hospital. The detection of respiratory pathogens was done using in-house TaqMan real-time PCR assays, and semi-quantitative results were reported based on the cycle threshold value (Ct-value). A high viral load was defined as a Ct-value <28, a medium viral load with Ct-values of 28–35, and a low viral load with Ct-values >35. Ct-values above 42 were regarded as virus-negative. In-house real-time PCR panels included analysis for human adenovirus, human bocavirus, human coronavirus OC43, NL63, 229E, human enterovirus, human parechovirus, HMPV, influenza A virus, influenza B virus, parainfluenza virus types 1–4, RSV, human rhinovirus, *Bordetella pertussis*, *Chlamydophila pneumoniae* and *Mycoplasma pneumonia* [35]. Conventional viral cultures were also performed. The detection of HMPV as a single virus in the NPA is named single HMPV, while HMPV co-detected with ≥1 other virus is named HMPV with co-detection. Similar expressions are also used for RSV. The HMPV-positive specimens were genotyped by real-time PCR and DNA sequencing using primers targeting the F-gene of HMPV [11]. A 527-bp amplicon was sequenced using a Big Dye Terminator v.3.1 cycle sequencing kit (Applied Biosystems) according to the manufacturer’s instructions. Sequences were analyzed on an ABI 3130XL (Applied Biosystems). Genotypes were determined by comparing sequenced data with the nucleotide BLAST database (www.ncbi.nlm.nih.gov/BLAST/). Some of the NPA were not type-able due to a low viral load, and others were empty after regular PCR tests.

Blood samples were collected to measure the concentration of C-reactive protein (CRP) in mg/L and white blood cell count x 10^9^/L.

### Statistical Analyses

To compare categorical variables, we used the χ² or Fisher exact tests when expected values were <5. Means of normally distributed continuous variables were compared by Student t-test or ANOVA, and non-normally distributed continuous or ordinal variables were compared by use of Mann-Whitney *U* or Kruskal-Wallis tests. We compared HMPV and RSV using stratified analyses among those with single virus infections and those with viral co-detections. Children with both HMPV and RSV were excluded from these analyses. Due to significant age differences between HMPV- and RSV-infected children, we also used a stratification strategy to control for age. We analyzed risk factors for severe disease in HMPV- and RSV-infected children separately, using logistic regression. We dichotomized the combined disease severity score, defining cases as a score ≥2 and controls as a score <2, and used this as a binary outcome in the logistic regression analyses. We predefined factors related to exposure and outcome to be age, prematurity, ≥1 chronic disease, viral co-detection and Ct-values. All factors were entered into full multivariable logistic regression models. Final models for HMPV and RSV, respectively, were obtained by stepwise removing factors with *P* > 0.1. Testing for a possible covariance between variables such as prematurity and ≥1 chronic disease showed that both variables could be entered into the same models, but that it was not possible to include specific chronic diseases. The results from the final models were presented as odds ratios (OR) with 95% confidence intervals (CI) and *P*-values, and used for inference.

Missing data has been specified in Fig 1 and tables, and no imputation of missing data has been done. *P*-values < 0.05 (two-sided) were considered as statistically significant, and IBM SPSS Statistics 22 were used in all analyses.

## Results

During nearly 9 years, we included 3,214 children out of 3,932 (81.7%) with acute RTI into our main study cohort (Fig 1). Among the 56.5% (1816/3214) of hospitalized children with LRTI, HMPV was detected in 9.4% (171/1816) and RSV in 47.3% (859/1816), while 0.6% (11/1816) had both HMPV and RSV (Fig 1). In total, 1,041 HMPV- and RSV-infected children were included in the present study (Fig 1). Their median age was 8.7 months (range 0.3–189.1 months) and the majority was younger than 5 years old (97.9%, 1019/1041). NPA were collected from 85.7% (892/1041) of these children within 24 hours after presentation, and within 48 hours in 96.0% (999/1041).

### Children with LRTI and HMPV

Tables 1–3 and S1 Table summarize baseline characteristics and details of the hospital courses among HMPV-infected children (n = 171). The children had a median age of 17.2 months and the majority were boys (59%). Nearly every fourth child was born premature. Every third child had ≥1 chronic disease. Asthma was the most common chronic disease at 20%, while 13% had other chronic airway diseases. The children presented at the hospital at a median of 4.0 days after onset of the symptoms, which most often were a cough (91%) and fever (88%). Symptoms of breathing difficulties and clinical signs of respiratory difficulty, such as chest retractions and tachypnea, appeared in half or more. The median peak CRP level was slightly elevated at 35 mg/L, whereas one-third had otitis media. Sixty-nine children had bronchiolitis (40%), which was slightly more frequent than pneumonia, appearing in 61 (36%). Obstructive bronchitis, asthma exacerbation and unspecified LRTI were less common. The majority of the patients received several treatment modalities, in particular inhalations (91%) and oxygen (60%), while fewer received antibiotics (39%) and corticosteroids (33%). Twenty-three (13%) children received respiratory support, among whom the majority were admitted to the PICU. The median length of stay at the hospital was 4.0 days, and the median severity score was 1.0. HMPV was detected as a single virus in 106 out of 171 (62%) children, while 65 (38%) had HMPV with a co-detection of one or more viruses (rhinovirus (n = 27), enterovirus (n = 22), parainfluenza virus 1–4 (n = 9), human bocavirus (n = 8), adenovirus (n = 6), human parechovirus (n = 3) and influenza A and B viruses (n = 4)). All baseline characteristics, symptoms, clinical findings, diagnoses and disease severity measures, such as a need for oxygen, length of hospital stay, a need for respiratory support and admission to the PICU, appeared at similar rates among both the children with a single virus infection and those with HMPV co-detection. Hence, the combined disease severity score was also equal.

**Table 1:** Baseline Characteristics and Medical history in Hospitalized Children with Lower Respiratory Tract Infection, by Virus Type (HMPV vs RSV) and Infection Status (single virus infection vs co-detection).

	HMPV				RSV				HMPV vs RSV, *P*	
	Total (n = 171)	Single (n = 106)	Co-detection (n = 65)	*P*	Total (n = 859)	Single (n = 540)	Co-detection (n = 319)	*P*	Single (n = 646)	Co-detection (n = 384)
**Age, months**	17.2 (8–29)	14.7 (8–25)	18.5 (9–29)	0.253	7.3 (2–17)	5.4 (1–14)	12.5 (4–20)	<0.001	<0.001	<0.001
**Age < 6 months**	29 (17)	22 (21)	7 (11)	0.433^a^	382 (44)	283 (52)	99 (31)	<0.001^a^	<0.001^a^	0.001^a^
**Age 6–11 months**	34 (20)	20 (19)	14 (22)		153 (18)	96 (18)	57 (18)			
**Age 12–23 months**	54 (32)	31 (29)	23 (35)		215 (25)	101 (19)	114 (36)			
**Age 24–59 months**	43 (25)	25 (24)	18 (28)		98 (11)	52 (10)	46 (14)			
**Age ≥ 60 months**	11 (6)	8 (8)	3 (5)		11 (1)	8 (1)	3 (1)			
**Gender, male**	101 (59)	64 (60)	37 (57)	0.656	477 (56)	289 (54)	188 (59)	0.123	0.195	0.764
**≥ 1 siblings**	124/163	77/102	47/61	0.821	612/821	385/514	227/307	0.760	0.900	0.611
	(76)	(75)	(77)		(75)	(75)	(74)			
**Day care**	67/170	36/105	31	0.082	254/858	123/539	131	<0.001	0.013	0.324
	(39)	(34)	(48)		(30)	(23)	(41)			
**Prematurity**^b^	39 (23)	22 (21)	17 (26)	0.414	117 (14)	64 (12)	53 (17)	0.049	0.014	0.069
**Asthma**	35 (20)	20 (19)	15 (23)	0.508	97 (11)	46 (9)	51 (16)	<0.001	0.001	0.167
**Other chronic airway disease**	22 (13)	16 (15)	6 (9)	0.266	23 (3)	16 (3)	7 (2)	0.500	<0.001	0.012
**Heart disease**	12 (7)	9 (8)	3 (5)	0.539	28 (3)	21 (4)	7 (2)	0.177	0.072	0.383
**Epilepsy**	7 (4)	5 (5)	2 (3)	0.710	11 (1)	7 (1)	4 (1)	1.0	0.033	0.269
**Cerebral palsy**	14 (8)	11 (10)	3 (5)	0.182	13 (2)	9 (2)	4 (1)	0.777	<0.001	0.098
**Other chronic disease**^c^	15 (9)	9 (8)	6 (9)	0.868	38 (4)	19 (4)	19 (6)	0.093	0.033	0.404
**≥ 1chronic disease**	59 (35)	36 (34)	23 (35)	0.849	150 (17)	81 (15)	69 (22)	0.013	<0.001	0.018

Data presented as absolute numbers and percent in parenthesis, except from “Age, months”, which is median and interquartile range (IQR) in parenthesis. Fractions are provided when sample size deviates from the given value.

^a^Comparing all age categories.

^b^Children born < 36 gestational weeks.

^c^Among single HMPV-infected as retinopathy (n = 1), endocrine disorder (n = 1), Down syndrome (n = 2), hereditary essential tremor (n = 1), congenital malformations (n = 2), muscular and neuromuscular disorder (n = 1+1). Among HMPV with co-detection as Down syndrome (n = 2), gastrointestinal disease (n = 2), endocrine disorder (n = 1) and neuromuscular disorder (n = 1). Among single RSV-infected as congenital malformations (n = 4), endocrine disorders (n = 2), Down syndrome and other syndromes (n = 4+6), Hemophilia A (n = 1) and gastrointestinal disease (n = 2). Among RSV with co-detection as unspecified psychomotor retardation (n = 3), endocrine disorders (n = 2), Down syndrome and other syndromes (n = 4+2), neuromuscular disorder (n = 1), long QT syndrome (n = 1), congenital malformations (n = 1), gastrointestinal and urinary tract disease (n = 4+1).

HMPV indicates human metapneumovirus; RSV, respiratory syncytial virus.

**Table 2:** Clinical Details in Hospitalized Children with Lower Respiratory Tract Infection (LRTI), by Virus Type (HMPV vs RSV) and Infection Status (single virus infection vs co-detection).

	HMPV				RSV				HMPV vs RSV, *P*	
	Total (n = 171)	Single (n = 106)	Co-detection (n = 65)	*P*	Total (n = 859)	Single (n = 540)	Co-detection (n = 319)	*P*	Single (n = 646)	Co-detection (n = 384)
**Tachypnea (admission)**	83/165 (50)	52/102 (51)	31/63 (49)	0.825	470/809 (58)	281/504 (56)	189/305 (62)	0.083	0.377	0.060
**Oxygen < 93% (admission)**	43/164 (26)	26/101 (26)	17/63 (27)	0.860	162/842 (19)	95/530 (18)	67/312 (21)	0.207	0.067	0.339
**Peak temp.**^a^, **°C, median**	38.6	38.6	38.6	0.824	38.2	38.1	38.5	<0.001	<0.001	0.288
**(IQR)**	(37.8–39.6)	(37.8–39.5)	(37.9–39.7)		(37.6–39.2)	(37.5–39.0)	(37.7–39.4)			
**Peak CRP**^b^, **median (IQR)**	35 (10–83)	35 (10–88)	26 (10–75)	0.449	19 (6–55)	15 (5–45)	31 (8–70)	<0.001	<0.001	0.668
**Peak WBC**^c^, **mean (SD)**	11.9 (4.7)	11.7 (4.7)	12.8 (5.4)	0.478	12.0 (5.1)	11.8 (4.9)	12.4 (5.4)	0.126	0.767	0.797
**Chest X-ray, abnormal**	120	74	46		445	263	182			
**Perihilar infiltrates /hyperinflation/atelectasis**	59 (49)	38 (51)	21 (46)	0.544^d^	259 (58)	164 (62)	95 (52)	0.033^d^	0.088^d^	0.428^d^
**Localized/lobular infiltrates/ pleural effusion**	61 (51)	36 (49)	25 (54)		186 (42)	99 (38)	87 (48)			
**Ct < 28**	92 (54)	56 (53)	36 (55)	0.743*	712/847 (84)	458/533 (86)	254/314 (81)	0.154*		
**Ct 28–35**	62 (36)	38 (36)	24 (37)		117/847 (14)	65/533 (12)	52/314 (17)			
**Ct > 35**	17 (10)	12 (11)	5 (8)		18/847 (2)	10/533 (2)	8/314 (3)			
**Inhalations**	155 (91)	94 (89)	61 (94)	0.260	824/858 (96)	521 (96)	303/318 (95)	0.385	0.001	0.543
**Antibiotics**	66 (39)	40 (38)	26 (40)	0.768	216/849 (25)	126/534 (24)	90/315 (29)	0.108	0.002	0.069
**Intravenous fluid**	44 (26)	32 (30)	12/64 (19)	0.099	179/848 (21)	109/533 (20)	70/315 (22)	0.541	0.027	0.539
**Nasogastric feed tube**	37 (22)	26/104 (25)	11/63 (17)	0.255	207/856 (24)	151/538 (28)	56/318 (18)	<0.001	0.522	0.977
**Corticosteroids**	57 (33)	37/104 (36)	20/64 (31)	0.565	219/850 (26)	115/536 (21)	104/314 (33)	<0.001	0.002	0.771
**Otitis media**	56 (33)	37 (35)	19 (29)	0.443	194 (23)	114 (21)	80 (25)	0.179	0.002	0.485
**Pneumonia**	61 (36)	36 (34)	25 (38)	0.619§	187 (22)	99 (18)	88 (28)	<0.001§	<0.001§	0.160§
**Bronchiolitis**	69 (40)	42 (40)	27 (42)		554 (64)	383 (71)	171 (54)			
**Bronchitis/unspec. LRTI**	17 (10)	13 (12)	4 (6)		28 (3)	18 (3)	10 (3)			
**Asthma exacerbation**	24 (14)	15 (14)	9 (14)	90 (10)	40 (7)	50 (16)	50 (16)			

Data presented as absolute numbers and percent in parenthesis, if otherwise not specified. Fractions are provided when sample size deviates from the given values.

^a^Temperature sampled from 168 HMPV (104 single and 64 co-detected) and from 794 RSV (499 single and 295 co-detected).

^b^CRP, C-reactive protein in mg/L, sampled from 170 HMPV (105 single and all 65 co-detected) and from 825 RSV (520 single and 305 co-detected).

^c^WBC, White blood cell count, sampled from 161 HMPV (102 single and 59 co-detected) and from 801 RSV (500 single and 301 co-detected).

^d^Comparing perihilar infiltrates/hyperinflation/atelectasis with localized/lobular infiltrates/pleural effusions.

*Comparing the three Ct, cycle threshold, categories.

^§^Comparing the four LRTI groups (pneumonia, bronchiolitis, obstructive bronchitis/unspecified LRTI and asthma exacerbation).

HMPV indicates human metapneumovirus; RSV, respiratory syncytial virus; IQR, interquartile range.

**Table 3:** Disease Severity measures in Hospitalized Children with Lower Respiratory Tract Infection, by Virus Type (HMPV vs RSV) and Infection Status (single virus infection vs co-detection).

	HMPV				RSV				HMPV vs RSV, *P*	
	Total (n = 171)	Single (n = 106)	Co-detection (n = 65)	*P*	Total (n = 859)	Single (n = 540)	Co-detection (n = 319)	*P*	Single (n = 646)	Co-detection (n = 384)
**Oxygen treatment, any**	102 (60)	64 (60)	38 (58)	0.804	542/857 (63)	351/539 (65)	191/318 (60)	0.138	0.351	0.810
**Oxygen (days), median (IQR)**^a^	4.0 (2.0–6.0)	3.0 (2.0–6.0)	4.0 (2.0–6.5)	0.571	3.0 (2.0–5.0)	3.0 (2.0–5.0)	3.0 (1.5–4.5)	0.572	0.073	0.022
**Resp. support, any**	23 (13)	15 (14)	8 (12)	0.732	108 (13)	77 (14)	31 (10)	0.056	0.977	0.529
**Resp. support, non-invasive**	21 (12)	14 (13)	7 (11)	0.637	105 (12)	75 (14)	30 (9)	0.053	0.852	0.734
**Resp. support, invasive**	8 (5)	5 (5)	3 (5)	1.0	12 (1)	11 (2)	1 (0)	0.038	0.160	0.016
**PICU admission**	20 (12)	14 (13)	6 (9)	0.432	90 (10)	65 (12)	25 (8)	0.052	0.737	0.707
**Length of stay, median (IQR)**	4.0 (2.0–6.0)	4.0 (2.8–6.0)	4.0 (2.0–6.0)	0.860	4.0 (2.0–6.0)	4.0 (2.0–6.0)	4.0 (2.0–6.0)	0.654	0.940	0.969
**Length of stay ≥ 6 days**	49 (29)	30 (28)	19 (29)	0.896	238 (28)	149 (28)	89 (28)	0.923	0.881	0.828
**Severity score, median (IQR)**	1.0 (0.0–2.0)	1.0 (0.0–2.0)	1.0 (0.0–2.0)	0.885	1.0 (0.0–2.0)	1.0 (0.0–2.0)	1.0 (0.0–2.0)	0.191	0.496	0.951
**Severity score ≥ 2**	48 (28)	30 (28)	18 (28)	0.931	242 (28)	158 (29)	84 (26)	0.357	0.843	0.821

Data presented as absolute numbers and percent in parenthesis, if otherwise not specified. Fractions are provided when sample size deviates from the given values.

^a^Data from 99 hMPV-infected

(61 single and 38 co-detected) and 525 RSV-infected (340 single and 185 co-detected).

HMPV indicates human metapneumovirus; RSV, respiratory syncytial virus; IQR, interquartile range; PICU, pediatric intensive care unit.

### Comparison of Children Infected with Different HMPV Genotypes

HMPV genotypes were available from 147 out of 171 (86%) children (S2 Table). Genotype B was most frequent, detected in 80 NPA (54%), of which 26 were B1 and 54 were B2. Genotype A was detected in 67 NPA (46%), of which 12 were A2a, 28 were A2b and 27 were A2 (unassigned). No samples were positive for A1. There were no differences between infections elicited by genotypes A and B in demographic characteristics, medical history and disease severity measures, as expressed by single variables and the combined disease severity score (S2 Table), though with one exception: Children infected with genotype B more often received respiratory support compared to children with genotype A (14/80, 18%, vs. 4/67, 6%, *P* = 0.034). However, when we only included single HMPV-infected in this analysis (n = 90), this difference disappeared (genotype B: 7/47, 15% vs. genotype A: 4/43, 9%, *P* = 0.419). Finally, when we compared subtypes B1 vs. B2, and A2a vs. A2b vs. A2 (unassigned), both as single virus infections and co-detections, no differences were found in disease severity score (data not shown).

### Children with LRTI and RSV

Tables 1–3 and S1 Table summarize baseline characteristics and details of the hospital courses among RSV-infected children (n = 859). The children had a median age of 7.3 months, and the majority were boys (56%). Bronchiolitis was the most common diagnosis, appearing in nearly two-thirds, and approximately one-fifth had pneumonia. Single virus infection with RSV appeared in 540 out of 859 children (63%), while 319 out of 859 (37%) had RSV with the co-detection of other viruses (rhinovirus (n = 178), human parechovirus (n = 54), human bocavirus (n = 53), coronavirus (n = 46), adenovirus (n = 28), parainfluenza virus 1–4 (n = 12) and influenza A and B viruses (n = 11). Children with single virus infection were younger compared to those with RSV and co-detection (median 5.4 vs. 12.5 months, *P* < 0.001). Children with RSV infection and co-detection more often had fever before admission, whereas other symptoms appeared at similar rates in both RSV groups. Furthermore, more children with RSV and co-detection were born premature and had ≥ 1 chronic disease; they also developed a higher peak temperature, higher peak CRP levels, more often had pneumonia and less often had bronchiolitis. There was a tendency that less children with RSV and co-detection needed respiratory support and admission to the PICU, whereas the need for oxygen, length of hospital stay and the combined disease severity score did not differ between the two RSV groups (Table 3).

### Children Infected with Both HMPV and RSV

Eleven hospitalized children had both HMPV and RSV (Fig 1). They were diagnosed with pneumonia (n = 6), bronchiolitis (n = 2), asthma exacerbation (n = 2) and unspecified LRTI (n = 1), and their median age was 24.3 months. The median length of stay was five days (IQR 2.0–8.0) and the severity score median 1.0 (IQR 0.0–2.0). Seven out of 11 (64%) needed oxygen, but no received respiratory support. There were no differences in length of stay, need for oxygen and the combined disease severity score when comparing children with both HMPV and RSV (n = 11) with the entire groups of children with HMPV and RSV (n = 171 and n = 859, respectively).

### Comparison of Children with LRTI Due to HMPV and RSV

**Fig 2:**
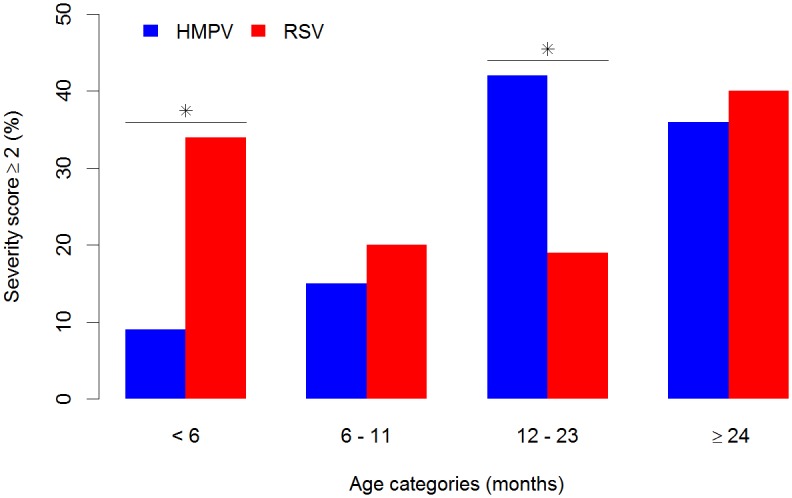
Proportions of children (%) with severe lower respiratory tract infection, severity score ≥ 2, among single virus-infected children with HMPV (blue) (n = 106) and RSV (red) (n = 540), according to age categories. Asterisk indicates significant differences (*P* < 0.05).

Tables 1–4 show comparisons of baseline characteristics, medical details and details of the hospital courses of the children with HMPV and RSV. First, we compared single virus-infected children with HMPV and RSV (Table 1). The single HMPV-infected children were older (14.7 months vs. 5.4 months, *P* < 0.001), more often were born premature and more often had chronic diseases including asthma, other chronic airway diseases, epilepsy and cerebral paresis, compared to children with a single RSV infection (Table 1). Age-stratified analyses showed that these differences primarily appeared in children aged 12–23 months (prematurity) and 6–11 months (chronic diseases), respectively (Table 4). More children with a single HMPV infection had otitis media and pneumonia, and were treated with antibiotics and corticosteroids (Table 2). In contrast, more children with a single RSV infection had bronchiolitis and received inhalations (Table 2). However, when we controlled for age we found no significant differences in the distribution of diagnoses (Table 4). Interestingly, among children aged <6 months, nine out of 10 children with HMPV, as well as those with RSV, had bronchiolitis (Table 4). Children with a single HMPV infection had a higher peak temperature and higher peak CRP levels, but adjusted for age, these differences also disappeared (Tables 1 and 4). When we compared the entire single virus-infected groups with HMPV and RSV, there were no differences in disease severity, i.e. there were no differences in need of oxygen and respiratory support, PICU admission and length of hospital stay. The combined disease severity scores were also equal (Table 3). However, age was a strong factor for explaining the disease severity (Table 4 and Fig 2). The effect of age differed among HMPV and RSV infected children. In the youngest age group of <6 months old, the children with HMPV infection had a milder disease because they less frequently needed oxygen, and they had a shorter hospital stay and a lower severity score (Table 4 and Fig 2). On the other hand, the single HMPV-infected children in the age group of 12–23 months had a more severe disease than the children with RSV; they were treated more days with oxygen, more often received respiratory support, more often were admitted to the PICU, had a longer hospital stay and more often had a severity score ≥2 (Table 4 and Fig 2).

**Table 4:** Clinical Characteristics in Hospitalized children with Lower Respiratory Tract Infection (LRTI), by Age and Single Virus Type (HMPV: n = 106 and RSV: n = 540).

	< 6 months			6–11 months			12–23 months			≥ 24 months		
	HMPV (n = 22)	RSV (n = 283)	*P*	HMPV (n = 20)	RSV (n = 96)	*P*	HMPV (n = 31)	RSV (n = 101)	*P*	HMPV (n = 33)	RSV (n = 60)	*P*
**Premature born**^a^	2 (9)	24 (8)	1.0	3 (15)	12 (13)	0.721	11 (35)	13 (13)	0.004	6 (18)	15 (25)	0.452
**≥ 1 chronic disease**	0 (0)	12 (4)	1.0	7 (35)	7 (7)	0.003	12 (39)	29 (29)	0.293	17 (52)	33 (55)	0.747
**Peak temp.**^b^, **median**	37.9	37.8	0.431	38.4	38.4	0.692	39.3	39.0	0.113	38.8	39.0	0.567
**(IQR)**	(37.4–38.6)	(37.4–38.2)		(37.6–39.9)	(37.9–39.2)		(38.3–39.8)	(38.1–39.6)		(38.0–39.5)	(38.0–39.7)	
**Peak CRP**^c^, **median**	18	9	0.219	27	15	0.270	31	32	0.987	76	42	0.094
**(IQR)**	(4–45)	(0–27)		(8–95)	(6–55)		(8–82)	(10–75)		(28–118)	(14–96)	
**Otitis media**	2 (9)	28 (10)	1.0	7 (35)	33 (34)	0.957	13 (42)	38 (38)	0.666	15 (45)	15 (25)	0.043
**Pneumonia**	1 (5)	25 (9)	1.0*	7 (35)	15 (16)	NA§	13 (42)	29 (29)	0.373#	15 (45)	30 (50)	0.675†
**Bronchiolitis**	20 (91)	256 (90)		8 (40)	69 (72)		14 (45)	58 (57)		0 (0)	0 (0)	
**Other diagnoses**^d^	1 (5)	2 (1)		5 (25)	12 (13)		4 (13)	14 (14)		18 (55)	30 (50)	
**Oxygen treatment**	4 (18)	177 (63)	<0.001	12 (60)	55/95 (58)	0.862	25 (81)	71 (70)	0.258	23 (70)	48 (80)	0.263
**Oxygen days**ǂ, **median**	3.0	3.0	0.888	2.0	2.5	0.670	4.0	2.0	0.010	4.5	4.0	0.822
**(IQR)**	(1.5–4.5)	(2.0–5.0)		(1.0–4.3)	(1.0–4.0)		(3.0–7.0)	(2.0–4.0)		(2.0–6.8)	(2.0–6.3)	
**Resp. support, any**	2 (9)	59 (21)	0.269	2 (10)	7 (7)	0.652	6 (19)	6 (6)	0.034	5 (15)	5 (8)	0.318
**Resp. support, non-invasive**	2 (9)	59 (21)	0.269	2 (10)	6 (6)	0.625	6 (19)	6 (6)	0.034	4 (12)	4 (7)	0.448
**Resp. support, invasive**	0 (0)	5 (2)	1.0	1 (5)	2 (2)	0.436	3 (10)	3 (3)	0.141	1 (3)	1 (2)	1.0
**PICU admission**	2 (9)	51 (18)	0.390	2 (10)	6 (6)	0.625	6 (19)	4 (4)	0.011	4 (12)	4 (7)	0.448
**Length of stay: median**	3.0	4.0	0.018	4.0	3.0	0.240	4.0	3.0	0.027	3.0	4.0	0.469
**(IQR)**	(1.8–4.0)	(3.0–6.0)		(3.0–5.0)	(2.0–5.0)		(3.0–8.0)	(2.0–5.0)		(2.0–7.5)	(3.0–7.0)	
**Length of stay ≥ 6 days**	3 (14)	81 (29)	0.130	2 (10)	21 (22)	0.356	13 (42)	23 (23)	0.036	12 (36)	24 (40)	0.730
**Severity score: median**	0.0	1.0	0.001	1.0	1.0	0.869	1.0	1.0	0.062	1.0	1.0	0.652
**(IQR)**	(0.0–0.3)	(0.0–2.0)		(0.0–1.0)	(0.0–1.0)		(1.0–2.0)	(1.0–1.0)		(0.0–2.0)	(1.0–2.0)	
**Severity score ≥ 2**	2 (9)	96 (34)	0.016	3 (15)	19 (20)	0.761	13 (42)	19 (19)	0.009	12 (36)	24 (40)	0.730

Data presented as absolute numbers and percent in parenthesis, if otherwise not specified. Fractions are provided when the sample size deviates from the given values.

^a^Born < 36 gestational weeks.

^b^Peak temperature in °C sampled from 104 HMPV-infected and 499 RSV-infected.

^c^CRP, C-reactive protein, sampled from 105 HMPV-infected and 520 RSV-infected.

^d^The sum of children with obstructive bronchitis/unspecified LRTI and asthma exacerbation.

*Comparing pneumonia and bronchiolitis.

^§^NA, not applicable, when comparing pneumonia, bronchiolitis and other diagnoses.

^#^Comparing pneumonia, bronchiolitis and other diagnoses.

^†^Comparing pneumonia and other diagnoses.

^ǂ^Days with oxygen sampled from 61 out of the 64 HMPV-infected, and from 340 out of the 351 RSV-infected, whom needed oxygen.

HMPV indicates human metapneumovirus; RSV, respiratory syncytial virus; IQR, interquartile range; PICU, pediatric intensive care unit.

We also compared the children with a co-detection of other viruses, in addition to HMPV and RSV (HMPV: n = 65 and RSV: n = 319) (Tables 1–3 and S1 Table). We retrieved the age difference seen in single virus-infected children, although it was less pronounced (HMPV: 18.5 months vs. RSV: 12.5 months, *P* < 0.001) (Table 1). More children with HMPV and co-detection had one or more chronic diseases, including chronic airway diseases other than asthma. There were no differences in peak temperature, peak CRP levels and LRTI diagnoses. The clinical courses also differed to some extent (Table 3). Although the same rate of children with HMPV and RSV co-detections needed oxygen, HMPV-positive children needed oxygen longer (*P* = 0.022). Invasive respiratory support was also given more often to HMPV-infected children (*P* = 0.016), whereas non-invasive respiratory support was given at similar rates (Table 3).

Two children died because of RTI during the entire nine-year-long study period. One was only 2.5 months old and had a single RSV infection. The other child was two years old and had a single HMPV infection. Both had severe comorbidities.

### Risk Factors for Severe Disease Due to HMPV and RSV

In the logistic regression analyses we found that age was strongly associated with disease severity (Table 5). In HMPV-infected children, age groups 12–23 months (OR = 3.01, *P* = 0.067) and ≥24 months (OR = 3.97, *P* = 0.021) were associated with the highest risk for severe disease, while in the youngest age group (<6 months) RSV-infected children had the highest risk for severe disease (OR = 2.11, *P* = 0.002). Prematurity was associated with higher risk in both HMPV- (OR = 3.36, *P* = 0.005) and RSV-infected children (OR = 1.58, *P* = 0.035). Chronic disease was also an important factor, being significantly associated with higher risk in RSV-infected children (OR = 2.26, *P* < 0.001) and close to significant in HMPV-infected children (OR = 2.22, *P* = 0.059). High viral load was associated with higher risk for severe disease in RSV-infected children only (OR = 7.91, *P* = 0.047) and not in those with HMPV. No significant interactions were present among variables included in the two final models, respectively. Finally, viral co-detection was not associated with increased risk for severe disease, and this factor was not included in the final models for HMPV and RSV. Other factors, not included in the predefined models, such as genotype (HMPV), gender, siblings and day care attendance, were also analyzed with logistic regression, but none of these factors yielded any significant contributions (data not shown).

**Table 5:** Results from Final Models^a^, presenting Odds Ratios (OR) with 95% Confidence Intervals (95% CI) and *P*-values, for the Associations between Risk Factors and Severe Lower Respiratory Tract Infection, by Virus Type (HMPV or RSV).

Risk factor	HMPV (n = 171)		RSV (n = 847)	
	OR (95% CI)	*P*	OR (95% CI)	*P*
Age < 6 months	1.16 (0.23–5.87)	0.853	2.11 (1.33–3.35)	0.002
Age 6–11 months	1.0		1.0	
Age 12–23 months	3.01 (0.93–9.75)	0.067	1.19 (0.70–2.00)	0.525
Age ≥ 24 months	3.97 (1.24–12.73)	0.021	1.34 (0.72–2.52)	0.360
Premature^b^ born	3.36 (1.44–7.83)	0.005	1.58 (1.03–2.42)	0.035
Not premature born	1.0		1.0	
≥ 1 chronic disease	2.22 (0.97–5.09)	0.059	2.26 (1.44–3.54)	< 0.001
No chronic disease	1.0		1.0	
Ct^c^ < 28	…		7.91 (1.03–61.05)	0.047
Ct 28–35	…		5.33 (0.67–42.59)	0.115
Ct > 35	…		1.0	

^a^Final binary logistic regression models, in which HMPV and RSV are analyzed separately. Full models included all factors as age, prematurity, chronic disease, Ct-values and viral co-detection. Factors were stepwise removed when *P* > 0.1.

^b^Born < 36 gestational weeks.

^c^Cycle threshold values, a measure of viral load. This factor was not included in the final model for HMPV.

HMPV indicates human metapneumovirus; RSV, respiratory syncytial virus.

## Discussion

The present data from our large population-based study collected during a nearly 9-year-long period show that LRTI with HMPV clinically manifests itself independently of the co-detection of other viruses, and does not differ in relation to HMPV genotypes. Furthermore, clinical manifestations and final diagnoses in children with HMPV and RSV LRTI are quite similar. However, the clinical course varies in relation to age, and the age effect differed among single virus HMPV- and RSV-infected children. Lastly, our data confirm that hospitalized children born preterm and children with chronic diseases have an increased risk of developing severe LRTI among HMPV- and RSV-infected.

Using a broad panel of sensitive nucleic-acid based viral tests, we detected more than one virus in 38% of the children with LRTI and HMPV. All patient characteristics, including rates of prematurity and chronic diseases, clinical manifestations and clinical courses were surprisingly equal in those with single HMPV infection and HMPV with viral co-detection. On this basis, and on observations done by others [14,17,23], it seems evident to conclude that viral co-detections in HMPV-infected children usually have no cumulative clinical effects to that of HMPV alone. Our population-based data also clearly shows that HMPV/RSV-coinfection is a rare event, and is usually not associated with increased severity, as has been suggested from smaller studies based on selected groups of children [21,22].

We detected a broad spectrum of HMPV genotypes (A2, A2a, A2b, B1 and B2), but no samples were positive for genotype A1. In other studies, genotype A1 has also been the most seldom genotype detected [13–15,17,36]. Our data clearly show that the present HMPV genotype variations do not relate to any particular premorbid condition, and causes infection with quiet similar clinical manifestations and outcomes in children. Consequently, our findings confirm results from previous studies with smaller sample sizes [13–15,36,37]. However, Schuster et al. [17] genotyped 192 HMPV-positive in children <2 years old in Jordan enrolled during three years, and found that HMPV genotype A infection was associated with an increased need for oxygen compared to genotype B infection. In another study, including 68 HMPV-positive hospitalized children <3 years of age enrolled during four winter seasons in Canada by Papenburg et al. [18], infection with HMPV genotype B was associated with either an increased oxygen need, PICU attendance or a hospitalization >5 days. The diverging findings in these two studies compared to our study may be explained by different age of the included children, and because the outcome “severe disease” in the two other studies probably included less ill children than in our study. Furthermore, naturally occurring genotype variations over shorter time intervals may also increase the risk of random findings.

The most prominent factor differing between HMPV and RSV in the present study was the difference in age distributions, which has been observed before [18,23,29,38,39]. Two-thirds with HMPV infection were 1 to 5 years old and less than one-fifth was <6 months old, and nearly half of RSV-infected children were <6 months old. In addition, we confirmed findings from previous studies that more HMPV-infected children were preterm born [18,29] and had a chronic disease [2,32]. On the other hand, HMPV and RSV in many ways caused a quite similar spectrum of LRTI types. Looking at the entire groups of children with HMPV and RSV infections, bronchiolitis was the most common diagnosis in both viruses, although a 50% higher rate was observed in children with RSV. By contrast, HMPV-infected children apparently had pneumonia and otitis media more often. However, these findings were confounded by age. In children <6 months old with a single virus infection, 90% of both viral infections were classified as bronchiolitis, and in children older than 6 months, no significant differences in bronchiolitis and pneumonia rates were found. In previous studies, it has been shown that temperature and CRP may increase to higher levels in children with HMPV infection than in those with RSV [3,38], but we could not confirm this after adjusting for age.

We found that disease severity was very similar when we compared the entire groups of children with HMPV and RSV, and others have also previously reported this [23,25,26]. However, age was strongly related to disease severity, and the age effect differed among single virus HMPV- and RSV-infected children. First of all, only one-fifth of the hospitalized children with single HMPV infection were younger than 6 months, compared to half of those with RSV. Secondly, HMPV infection was associated with a milder disease than RSV infection among children aged <6 months, as indicated by a less frequent need for oxygen, a shorter hospital stay and a lower severity score. Furthermore, the data provided evidence that in children aged 12–23 months old, HMPV infection may be more severe than RSV infection, with a longer need for oxygen treatment, more children in need of respiratory support, more children admitted to PICU, a longer hospital stay and a higher severity score. A possible explanation for these observations might have been that neonates attain higher concentrations of maternally derived protective antibodies against HMPV, as compared to RSV, during pregnancy and the first six months of life. However, data from a recent clinical study measuring HMPV and RSV antibody concentrations did not confirm this hypothesis [40]. Another explanation might have been that HMPV-infected children more often than children with RSV had a primary and potentially more severe infection among those children aged 12–23 months, but it was not possible for us to assess whether children had a primary or secondary infection. In general, clinical manifestations in children with airway infections are related to the net effect of physical and genetic factors, as well as viral- and immune-mediated reactions in the maturing child, which are strongly correlated to the child’s age [41,42]. We found that high RSV viral loads, but not high HMPV viral loads, were associated with severe disease. Thus, based on these clinical observations among our population of hospitalized children, it may be tempting to claim that RSV is a more potent virus than HMPV among infants, and that RSV infection more than HMPV is a virally driven disease. Recently, other researchers have published data supporting a similar assumption [43]. In accordance with our findings, Roussy et al. [37] found that HMPV viral loads were not associated with increased disease severity among hospitalized children (inpatients), but hospitalized patients had higher HMPV viral loads than outpatients [37]. It has also been shown by others that LRTI may be associated with higher HMPV viral loads than URTI [44]. For this reason, it seems that HMPV viral loads may relate to disease severity to a certain extent, but not among those with the most severe disease.

Most previous studies on risk factors for severe HMPV infections in children focused on age groups younger than 2–3 years old [17,18], high-risk patients [29] or for children admitted to PICU [33], and disease severity has been defined by the use of various outcome variables [17,18,29,33]. We included a population-based sample consisting of all children aged <16 years who were admitted with acute RTI, although the vast majority were aged <5 years. We used a compound severity score combining several outcome measures. Although this score has not been validated, it fit the routines at our department and rather rigorously defined severe disease, and provided reliable risk factor estimates. We confirmed that independent risk factors for both severe HMPV and RSV infections were the presence of chronic diseases and a history of prematurity. Children aged 12 to 23 months had a three-fold increased risk of developing severe HMPV infection, and those aged ≥24 months had a nearly four-fold increased risk. Among RSV-infected children, infants less than six months had a nearly double risk compared to older children. Having one or more chronic diseases doubled the risk in both virus types, but due to a significant co-variation, our data set could not be used to identify which chronic diseases more precisely increased the risk. Prematurity with a gestational age less than 36 weeks increased the risk of severe HMPV infection three-fold, as shown by others [31], and severe RSV infection for approximately 50%. However, prophylactic use of palivizumab in high-risk children may have confounded this risk estimation in relation to RSV. Hence, in hospitalized children, our data confirm the findings from other studies that particular age groups, prematurity and the presence of chronic diseases independently increase the risk of developing severe LRTI among children with HMPV infection [2,17,18,29,31–33] and RSV infection [18,24,27–30].

It is a strength of the present study that we prospectively enrolled children of all ages from the same county in Mid-Norway, and to the only existing hospital in this region during a nearly 9-year long period. NPA were taken from the majority of the admitted children, and 81.7% were included in the main study cohort. Moreover, we analyzed all NPA using a broad panel of sensitive virus tests during the entire period, which allowed us to examine viral co-detections thoroughly. Nonetheless, it may be a limitation that bacterial co-detections were not considered, but most children had low or moderately increased CRP values. Furthermore, during the entire study period almost all Norwegian children received conjugated pneumococcal vaccines, which has reduced the incidence of pneumococcal infections [45]. Although this does not completely exclude pneumococcal coinfection, at least HMPV- and RSV-infected children may have been similarly influenced. Diagnostic and work-up biases could have affected our results negatively, since the clinicians were not blinded for the NPA results, and because patients were not treated after a study protocol.

In conclusion, HMPV infections among hospitalized children with LRTI were manifested independently of viral co-detection and HMPV genotypes. HMPV and RSV infections differed clinically to a certain extent, and these differences were mostly related to age. Among single virus-infected children, HMPV-infected aged <6 months had a milder disease and those aged 12–23 months had more severe disease, than children with RSV. A history of prematurity and chronic disease increased the risk of severe LRTI among HMPV- and RSV-infected children.

## Supporting Information

S1 Table Symptoms, Clinical findings at Admission and Upper Respiratory Tract Infection Diagnoses in Hospitalized Children with Lower Respiratory Tract Infection, by Virus Type (HMPV vs RSV) and Infection Status (single virus infection vs co-detection). Data presented as absolute numbers and percent in parenthesis, except from symptom-days as median with interquartile range, IQR, in parenthesis. Fractions are provided when samples size deviates from the given value. *Data from 163 HMPV-infected (102 single and 61 co-detected) and 831 RSV-infected (519 single and 312 co-detected). HMPV indicates human metapneumovirus; RSV, respiratory syncytial virus.
(DOCX)

S2 Table Medical history, Clinical Details and Disease Severity measures in 147 children with Lower Respiratory Tract Infection (LRTI), by HMPV genotype A vs B. Data presented as absolute numbers and percent in parenthesis, if otherwise not specified. *CRP, C-reactive protein, sampled from all HMPV A and 79 HMPV B. †WBC, White blood cell count, sampled from 60 HMPV A and 78 HMPV B. ‡NA, not applicable, when comparing the three Ct categories. §Comparing the four LRTI groups (pneumonia, bronchiolitis, obstructive bronchitis/unspecified LRTI and asthma exacerbation). HMPV indicates human metapneumovirus; RSV, respiratory syncytial virus; IQR, interquartile range, GA, gestational age; Ct-values, cycle threshold values.
(DOCX)
