# Supplementary material for: Comparing Human Metapneumovirus and Respiratory Syncytial Virus: Viral Co-Detections, Genotypes and Risk Factors for Severe Disease
Source: PLoS One. 2017 Jan 17;12(1):e0170200. doi: 10.1371/journal.pone.0170200 (PMC5240941; doi:10.1371/journal.pone.0170200)
Supplement: S1 Table — Data presented as absolute numbers and percent in parenthesis, except from symptom-days as median with interquartile range, IQR, in parenthesis. Fractions are provided when samples size deviates from the given value. *Data from 163 HMPV-infected (102 single and 61 co-detected) and 831 RSV-infected (519 single and 312 co-detected). HMPV indicates human metapneumovirus; RSV, respiratory syncytial virus. (DOCX) [file pone.0170200.s001.docx]

| **S1 Table. Symptoms, Clinical findings at Admission and Upper Respiratory Tract Infection Diagnoses in Hospitalized Children with Lower Respiratory Tract Infection, by Virus Type (HMPV vs RSV) and Infection Status (single virus infection vs co-detection).** | | | | | | | | | | |
| --- | --- | --- | --- | --- | --- | --- | --- | --- | --- | --- |
|  | **HMPV** | | | | **RSV** | | | | **HMPV vs RSV, *P*** | |
|  | **Total**  **(n = 171)** | **Single**  **(n = 106)** | **Co-detection**  **(n = 65)** | ***P*** | **Total**  **(n = 859)** | **Single**  **(n = 540)** | **Co-detection**  **(n = 319)** | ***P*** | **Single**  **(n = 646)** | **Co-detection**  **(n = 384)** |
| Cough | 155 (91) | 93 (88) | 62 (95) | 0.095 | 762 (89) | 475 (88) | 287 (90) | 0.370 | 0.948 | 0.167 |
| Fever | 150 (88) | 93 (88) | 57 (88) | 0.993 | 591 (69) | 356 (66) | 235 (74) | 0.018 | <0.001 | 0.016 |
| Heavy breathing | 114 (67) | 70 (66) | 44 (68) | 0.824 | 651 (76) | 406 (75) | 245 (77) | 0.679 | 0.041 | 0.121 |
| Stuffed nose | 94 (55) | 57 (54) | 37 (57) | 0.688 | 564 (66) | 362 (67) | 202 (63) | 0.268 | 0.009 | 0.332 |
| Reduced appetite | 88 (51) | 61 (58) | 27 (42) | 0.042 | 519 (60) | 326 (60) | 193 (61) | 0.970 | 0.588 | 0.005 |
| Wheezing | 62 (36) | 37 (35) | 25 (38) | 0.639 | 396 (46) | 247 (46) | 149 (47) | 0.783 | 0.039 | 0.223 |
| Trout pain | 34 (20) | 21 (20) | 13 (20) | 0.976 | 164 (19) | 102 (19) | 62 (19) | 0.844 | 0.825 | 0.917 |
| Ear pain | 21 (12) | 13 (12) | 8 (12) | 0.993 | 70 (8) | 42 (8) | 28 (9) | 0.605 | 0.130 | 0.373 |
| Apnea | 1 (1) | 0 (0) | 1 (2) | 0.380 | 36 (4) | 25 (5) | 11 (3) | 0.404 | 0.023 | 0.699 |
| Symptom-days* before admission | 4.0 (3.0-5.0) | 4.0 (3.0-5.0) | 4.0 (2.0-5.0) | 0.252 | 4.0 (3.0-5.0) | 4.0 (3.0-5.0) | 4.0 (2.0-5.75) | 0.700 | 0.059 | 0.798 |
| Abnormal tympanic membrane | 55/169 (33) | 37/104 (36) | 18 (28) | 0.287 | 190 (22) | 111 (21) | 79 (25) | 0.151 | <0.001 | 0.621 |
| Pharyngitis | 57/169 (34) | 36/104 (35) | 21 (32) | 0.758 | 299 (35) | 178 (33) | 121 (38) | 0.140 | 0.743 | 0.392 |
| Rhinitis | 45/169 (27) | 28/104 (27) | 17 (26) | 0.912 | 280 (33) | 174 (32) | 106 (33) | 0.761 | 0.286 | 0.265 |
| Tonsillitis | 21/169 (12) | 14/104 (13) | 7 (11) | 0.606 | 70 (8) | 35 (6) | 35 (11) | 0.020 | 0.014 | 0.962 |
| Retractions at inspection | 115/170 (68) | 74/105 (70) | 41 (63) | 0.316 | 635 (74) | 404 (75) | 231 (72) | 0.439 | 0.353 | 0.131 |
| Expiratory wheezing (auscultation) | 69/170 (41) | 36/105 (34) | 33 (51) | 0.033 | 384 (45) | 243 (45) | 141 (44) | 0.820 | 0.043 | 0.332 |
| Rales (auscultation) | 70/170 (41) | 44/105 (42) | 26 (40) | 0.806 | 387 (45) | 256 (47) | 131 (41) | 0.071 | 0.301 | 0.873 |
| Crepitation (auscultation) | 59/170 (35) | 37/105 (35) | 22 (34) | 0.853 | 280 (33) | 169 (31) | 111 (35) | 0.290 | 0.428 | 0.883 |
| Otitis media | 56 (33) | 37 (35) | 19 (29) | 0.443 | 194 (23) | 114 (21) | 80 (25) | 0.179 | 0.002 | 0.485 |
| Rhino-pharyngitis | 97 (57) | 62 (58) | 35 (54) | 0.552 | 502 (58) | 307 (57) | 195 (61) | 0.219 | 0.755 | 0.272 |
| Tonsillitis | 21 (12) | 14 (13) | 7 (11) | 0.637 | 71 (8) | 35 (6) | 36 (11) | 0.013 | 0.017 | 0.904 |
| Acute laryngitis | 1 (1) | 1 (1) | 0 (0) | 1.0 | 5 (1) | 3 (1) | 2 (1) | 1.0 | 0.513 | 1.0 |

Data presented as absolute numbers and percent in parenthesis, except from symptom-days as median with interquartile range, IQR, in parenthesis. Fractions are provided when samples size deviates from the given value. *Data from 163 HMPV-infected (102 single and 61 co-detected) and 831 RSV-infected (519 single and 312 co-detected).

HMPV indicates human metapneumovirus; RSV, respiratory syncytial virus.
