# Supplementary material for: Comparing Human Metapneumovirus and Respiratory Syncytial Virus: Viral Co-Detections, Genotypes and Risk Factors for Severe Disease
Source: PLoS One. 2017 Jan 17;12(1):e0170200. doi: 10.1371/journal.pone.0170200 (PMC5240941; doi:10.1371/journal.pone.0170200)
Supplement: S2 Table — Data presented as absolute numbers and percent in parenthesis, if otherwise not specified. *CRP, C-reactive protein, sampled from all HMPV A and 79 HMPV B. †WBC, White blood cell count, sampled from 60 HMPV A and 78 HMPV B. ‡NA, not applicable, when comparing the three Ct categories. §Comparing the four LRTI groups (pneumonia, bronchiolitis, obstructive bronchitis/unspecified LRTI and asthma exacerbation). HMPV indicates human metapneumovirus; RSV, respiratory syncytial virus; IQR, interquartile range, GA, gestational age; Ct-values, cycle threshold values. (DOCX) [file pone.0170200.s002.docx]

| **S2** **Table.** **Medical history, Clinical Details and Disease Severity measures in 147 children with Lower Respiratory Tract Infection (LRTI), by HMPV genotype A vs B.** | | | |
| --- | --- | --- | --- |
|  |  |  |  |
|  | **HMPV A**  **(n = 67)** | **HMPV B**  **(n = 80)** | ***P*** |
| **Age, months, median (IQR)** | 14.7 (9-24) | 18.5 (7-33) | 0.295 |
| **Gender, male** | 40 (60) | 49 (61) | 0.848 |
| **Prematurity (GA < 36 weeks)** | 14 (21) | 17 (21) | 0.958 |
| **≥ 1 chronic disease** | 23 (34) | 24 (30) | 0.575 |
| **Peak CRP*, median (IQR)** | 29 (9-61) | 35 (10-79) | 0.511 |
| **Peak WBC†, mean (SD)** | 10.9 (4.3) | 12.5 (4.6) | 0.042 |
| **Ct < 28** | 42 (63) | 43 (54) | NA‡ |
| **Ct 28-35** | 21 (31) | 36 (45) |  |
| **Ct > 35** | 4 (6) | 1 (1) |  |
| **Viral co-detection** | 24 (36) | 33 (41) | 0.501 |
| **Pneumonia** | 20 (30) | 28 (35) | 0.336§ |
| **Bronchiolitis** | 34 (51) | 29 (36) |  |
| **Obstructive bronchitis and unspecific LRTI** | 5 (7) | 9 (11) |  |
| **Asthma exacerbation** | 8 (12) | 14 (18) |  |
| **Oxygen treatment, any** | 45 (67) | 45 (56) | 0.176 |
| **Respiratory support** | 4 (6) | 14 (18) | 0.034 |
| **PICU admission** | 4 (6) | 11 (14) | 0.121 |
| **Length of stay, median (IQR)** | 4.0 (3.0-6.0) | 4.0 (2.0-6.0) | 0.915 |
| **Severity score, median (IQR)** | 1.0 (0.0-2.0) | 1.0 (0.0-2.0) | 0.788 |
| **Severity score ≥ 2** | 18 (27) | 22 (28) | 0.931 |

Data presented as absolute numbers and percent in parenthesis, if otherwise not specified. *CRP, C-reactive protein,

sampled from all HMPV A and 79 HMPV B. †WBC, White blood cell count, sampled from 60 HMPV A and 78 HMPV B.

‡NA, not applicable, when comparing the three Ct categories. §Comparing the four LRTI groups (pneumonia, bronchiolitis,

obstructive bronchitis/unspecific LRTI and asthma exacerbation).

HMPV indicates human metapneumovirus; RSV, respiratory syncytial virus; IQR, interquartile range, GA, gestational age;

Ct-values, cycle threshold values.
